# Adaptation and validation of a protein intake screening tool for a UK adult population

**DOI:** 10.1017/jns.2022.96

**Published:** 2022-11-10

**Authors:** Esme R. Tuttiett, Elysa Ioannou, Hanneke A.H. Wijnhoven, Bernard M. Corfe, Elizabeth A. Williams

**Affiliations:** 1 Department of Oncology and Metabolism, The Medical School, The University of Sheffield, Sheffield S10 2RX, UK; 2 Sport and Physical Activity Research Centre, Sheffield Hallam University, Sheffield S10 2BP, UK; 3 Department of Health Sciences, Faculty of Science, and Amsterdam Public Health Research Institute, Vrije Universiteit Amsterdam, Amsterdam 1081 HV, The Netherlands; 4 Human Nutrition Research Centre, Faculty of Medical Sciences, Population Health Sciences Institute, Newcastle University, Newcastle NE2 4HH, UK

**Keywords:** Nutritional assessment, Protein intake, Rapid screening, Validation

## Abstract

Adequate dietary protein intake is important in human subjects for maintaining muscle turnover, determining the protein content of tissues and thus the preservation of muscle mass and function as we age. A screening tool to assess if an older individual is likely to have a lower dietary protein intake (predicted probability of protein intake ≤1⋅0 g/kg per d), has been developed for a Netherlands dietary profile, but this has not been validated in a UK population. This study aimed to adapt and then validate the protein screening tool for use in a UK population. Amendment of the tool was undertaken using data from UK BioBank and the UK National Diet and Nutrition Survey to reflect protein sources in the UK diet. Validation of the amended version of the protein screener screening tool was conducted using protein intake derived from a food frequency questionnaire (FFQ) in a sample of UK adults (*n* = 184) (age range 18–91 years) as the reference standard. Using the FFQ, 40 % of respondents (*n* = 74) reported a protein intake of ≤1⋅0 g per kg body mass. The discriminative accuracy of the amended screener was tested using receiver operating characteristic (ROC) curves. The area under the curve for the ROC was 0⋅731 (95 % CI 0⋅657, 0⋅805), indicating that the amended screener may be a valid tool to screen for individuals consuming ≤1⋅0 g/kg adjusted BM/d in an adult UK population. This protein screener tool is a potential method to screen individuals with a likelihood of habitually consuming protein intakes of ≤1⋅0 g/kg per d. Further validation is needed using a more robust dietary intake methodology and for specific groups, such as older adults. The screener may be applicable across healthcare, clinical and research applications.

## Introduction

The preservation of muscle mass and strength is vital for the maintenance of physical function and positive health outcomes in older age^(1,2)^. Sarcopenia is a disease defined by the loss of muscle mass and function, associated with aging^(3)^. The rate of loss of muscle mass accelerates during the fourth and fifth decades of life^(4)^. Ensuring protein intake is adequate throughout the life course may be beneficial as a preventative strategy for alleviating muscle loss during later life^(5)^. It is increasingly recognised that skeletal muscle plays an important role in health and disease prevention and dietary protein feeds into this through the influence it can have on whole body protein metabolism^(1)^. As such, having an understanding if protein intake is low, and responding to address this, could be potentially beneficial for preventing or delaying the onset of sarcopenia and metabolic diseases. The current UK Reference Nutrient Intake (RNI) for protein is 0⋅75 g per kg body mass/day (g/kg BM/d)^(6)^. Older individuals are less responsive to stimuli associated with muscle protein synthesis (MPS) although MPS anabolism remains stimulable, albeit in response to higher dosing^(7)^. Accordingly, the PROT-AGE study group^(8)^ and the European Society for Clinical Nutrition and Metabolism (ESPEN)^(9)^ recommend intakes of 1–1⋅2 g/kg BM/d for healthy older adults, up to 1⋅5  g/kg BM/dfor older people with acute or chronic disease and up to 2 g/kg per d for malnourished older adults.

Despite positive associations between dietary protein intake and muscle mass and function^(10,11)^, many protein intervention studies have yielded mixed results. Meta-analyses assessing benefits following protein supplementation interventions are equivocal^(12–15)^; however, in the included trials, most study participants already consumed protein at or above the recommended intake levels for protein. Trial participants may, therefore, not be representative of the general population, questioning the generalisability of these results. Indeed, in a study which performed a secondary analysis on two pre-existing dietary datasets for older adults, it was noted that 36 % of community-dwelling 65–89-year-olds, in South Yorkshire, UK, failed to meet current UK RNI levels (0⋅75 g/kg BM/d)^(16)^, with 85 % of this population falling short of the ESPEN protein recommendations for older adults (1⋅2 g/kg BM/d). Further to this, a cross-sectional analysis from five nutritional studies of older adults carried out in Europe, including data taken from responses to The Mini Nutritional Assessment, revealed that 74 % of individuals, who were otherwise classed as having a normal nutritional status, did not achieve the ESPEN protein intake recommendations^(17)^. Two recent dietary protein intervention studies have specifically recruited older adults with habitual low protein intakes (<1⋅0 g/kg adjusted body mass/d) and have reported beneficial effects of protein supplementation on lean body mass^(18)^ and physical performance^(19)^. This supports the hypothesis that those consuming less than the ESPEN recommendations for older adults^(9)^ may derive the greatest benefit from the elevation of intake. Dietary protein intake screening in practice would be advantageous in order to target dietetic or nutritional support for inadequate-protein consumers and to identify low protein consumers for intervention trials. An appropriate, first-step, screening tool that can quickly identify if an individual is likely to consume protein levels that are ≤1 g/kg BM/d could aid with the identification of those who may benefit from protein supplementation or intervention.

Current dietary assessment methods used to measure a person's nutrient intake are time-consuming to assess and analyse^(20)^. A short food questionnaire, the Protein Screener 55+ (Pro55+)^(21)^ was designed to screen for protein intakes ≤1⋅0 g/kg adjusted BM/d in community-dwelling older adults. It was externally validated retrospectively in an independent Dutch study sample and showed good discriminative ability compared to a full food frequency questionnaire (FFQ). The aim of this study is to adapt and validate this tool for use with an adult UK population (ProScreenerUK).

## Methods

### Adaptation of the protein screener tool

**Fig. 1.**
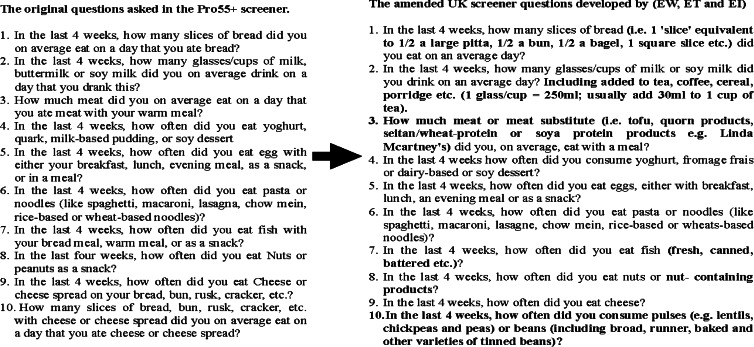
A demonstration of the original questions from the Dutch protein screener (left) and the adaptations that occurred to produce the amended questions used for the ProScreenerUK (right.) Changes to any of the questions asked in the UK version of the screener, compared to the Dutch version, have been highlighted in bold.

A protein screener tool was developed by Wijnhoven *et al.* in the Netherlands. This is a short food questionnaire, ten food questions in length, which was designed to screen for low protein intakes (≤1 g/kg BM/d.) The original questions were selected with the use of data from the Longitudinal Ageing Study Amsterdam (LASA) cohort study^(22)^. An external validation process was then carried out using data from the HEalthy LIfe in an Urban Setting (HELIUS) cohort study^(23)^. Amendments to the Dutch protein screener to reflect the UK diet were carried out via an iterative process conducted by the research team (EW, ET, EI), whilst also aiming to minimise changes. Firstly, common sources of protein-containing foods consumed in the UK and respective protein content of these foods were identified using UK BioBank and NDNS data^(24–26)^. This led to the replacement of a question on cheese consumption with one about the consumption of legumes and pulses. Meat substitutes were also included in the UK questionnaire and incorporated within the question on meat products. Further minor adjustments to the phrasing of the questions were made, but the total number of questions remained the same. The final amendments to the ten food intake questions are displayed in Fig. 1. The regression equation to calculate the predicted probability of protein intakes ≤1⋅0 g/kg adjusted BM/d was recalculated by replacing the ‘cheese question’ with the ‘legumes question’ and following the exact same procedures as described by Wijnhoven^(21)^ using the development LASA and validation HELSI samples (syntax and regression equation data available in Supplementary material.) The predicted probability of protein intakes for each participant was calculated by using this adapted regression equation. A predicted probability above 0⋅3 has been identified as the optimal cut-off within the Dutch sample as it best balances sensitivity and specificity, to screen for protein intakes ≤1⋅0 g/kg adjusted BM/d.

### Validation of the protein screener tool

#### Study design, sample and ethics

This was a cross-sectional study. Advertisement of the trial was via online adverts and direct e-mails to local clubs,community groups and the University of Sheffield's staff and student volunteer email list. The study was conducted during the COVID-19 pandemic and coincided with social distancing and lockdown restrictions (summer 2020). As a result, all recruitment, consent and interviews were undertaken remotely. Recruitment target numbers were a minimum of 163 participants. This was based on guidelines for sample size requirements for sensitivity and specificity analysis^(27)^. Calculations were based on developing a screening tool, and therefore prioritising sensitivity. Protein deficiency prevalence rates were estimated at 30 %^(16,28)^ and hypothesis null (H_0_) and hypothesis alternative (H_a_) were set at 0⋅5 and 0⋅7, respectively, based on area under the curve (AUC) targets for Receiver Operating Characteristic (ROC) curves. An AUC of >0⋅7 is considered as ‘fair’ and of >0⋅8 as ‘good' to establish ProScreenerUK as a screening tool^(29,30)^. There was no cap on recruitment numbers past the minimum value, as it has been stated larger sample sizes are optimal^(31)^. The recruitment window remained open from 01⋅06⋅20–20⋅07⋅20. Inclusion criteria: UK-dwelling adults aged 18 years+; exclusion criteria: Non-UK residents. A pilot questionnaire was completed in advance of the roll out of the questionnaire with ten subjects that led to amendments in the questionnaire. Given the wide age range that the sample represented we also aimed to recruit fifteen participants in each of the following age categories: 18–24, 25–34, 35–44, 45–54, 55–64, 65–74 and 75 years and recruitment strategies were targeted accordingly. Participants were asked to complete a three-part questionnaire, online, *via* googleforms. Participants unable to complete the study documentation online were able to do this via telephone interview. The questionnaire comprised a demographic data collection section (including age, height and weight), the amended protein screener (see final version in Fig. 1) and the 130-item EPIC FFQ^(32)^. Ethical approval for this study was granted by the University of Sheffield's Ethics Committee (Reference number: 032490).

#### Validation protocol

FETA nutritional analysis software^(33)^ was used to analyse the FFQs and to provide an estimate of daily protein intake (g/d). Relative protein intake (g/kg BM/d) was then calculated by dividing this absolute value by the body mass of each participant, if they had a ‘normal’ BMI (18⋅5–27 kg/m^2^.) For participants who were under- or over- weight according to BMI, body mass was first adjusted. This was based on the assumption that underweight individuals require more protein to build tissue and overweight and obese individuals require less protein per kg, due to excess mass carried as adipose tissue. Therefore, for participants whose BMI was >25 kg/m^2^ (≤70 years old) or >27 kg/m^2^ (>70 years old), the body mass (kg) applied corresponded to the BMI of 25 or 27 kg/m^2^ respectively. For participants whose BMI was <18⋅5 kg/m^2^ (≤70 years old) or <22⋅0 kg/m^2^ (>70 years old), the body mass (kg) applied corresponded to the BMI of 18⋅5 or 22 kg/m^2^, respectively^(21)^. This intake was then dichotomised into two categories based on whether the intake of protein was >1⋅0  g/kg adjusted BM/d or ≤1⋅0  g/kg adjusted BM/d.

### Statistical analysis

Data were checked for normality using a Shapiro–Wilk test. Sensitivity, Specificity, Positive Predictive Value (PPV) and Negative Predictive Value (NPV) were calculated for three cut-offs (>0⋅1, >0⋅2 and >0⋅3) of the predicted probability (see Table 2). The discriminative accuracy of the adapted regression equation was tested *v*. protein intake ≤1⋅0 g/kg adjusted BM/d (values of which were obtained from FFQ responses) using ROC curves. Youden Index scores (‘sensitivity + specificity – 1) were also calculated to aid with deciphering the optimal cut-off in this sample.

## Results

### Sample characteristics

**Fig. 2.**
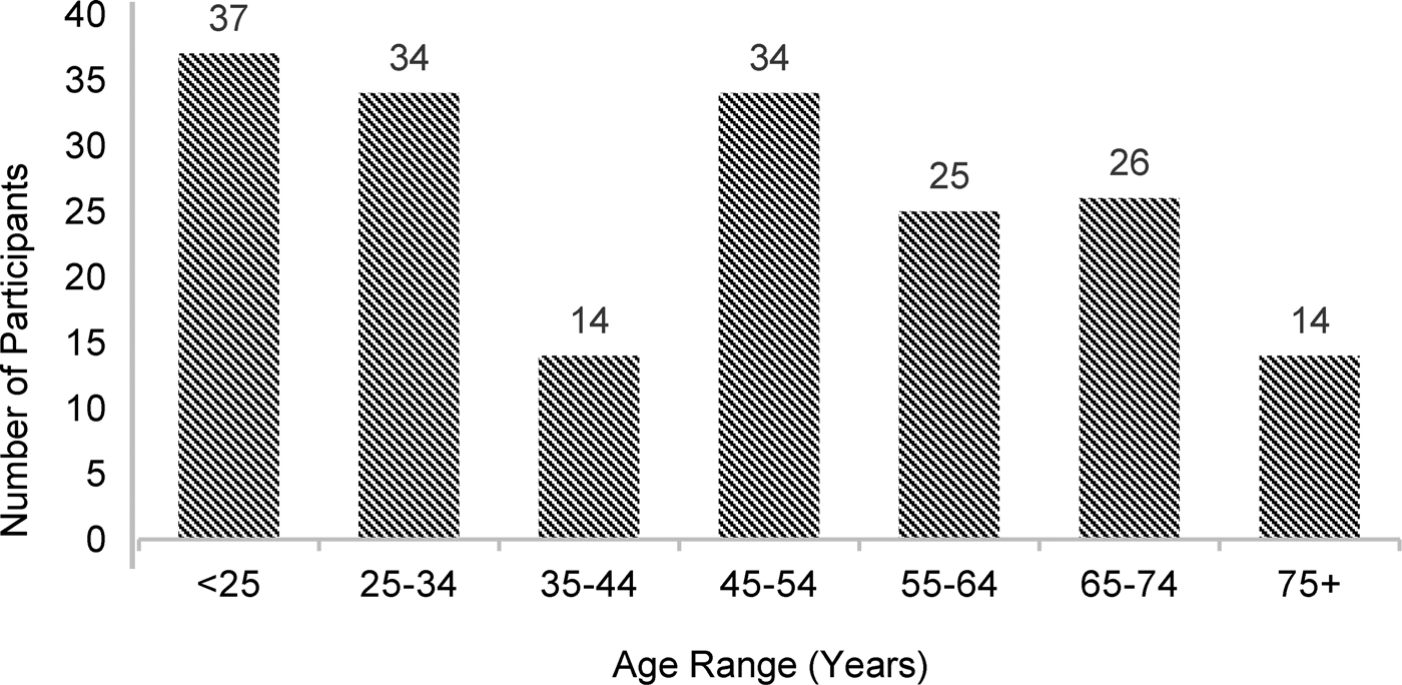
A breakdown of the number of the number participants who fell into each age category in the study.

Two-hundred-and-eight participants were recruited and completed the FFQ and protein screener questionnaire. Twenty-four participants were excluded from the analysis: reasons for exclusion were: had taken part in a pilot (11), were duplicates (6), were not UK dwelling (4) and did not complete the consent (3). A total of 184 were included in this analysis of whom 74 % (*n* = 137) were female. The mean (±sd) age (years) was 46 (±19) and the mean BMI (kg/m^2^) of the participants was 25⋅0 (±5). There was representation across a wide age range (18–91 years) (Fig. 2), with ninety-nine participants (54 %) over the age of 45.

### Dietary analysis

The mean (±sd) macronutrient intakes based on the FFQ responses for all participants are shown in Table 1. With respect to protein, 40 % (*n* = 74) had a protein intake ≤1⋅0 g/kg adjusted BM/d and 14 % (*n* = 27) had a protein intake of less than ≤0⋅75 g/kg BM/d.

**Table 1.** Average macronutrient composition, including protein adjusted for body mass, for all participants (*n*=184) and participants stratified by their protein intakes, from their habitual diet based on nutritional information obtained from FFQ data. Data presented as Mean (sd).

	Average intakes for all participants (*n* 184)	Average intakes for participants consuming ≤1 g/kg BM protein (*n* 74)	Average intakes for participants consuming >1 g/kg BM protein (*n* 110)
Energy (kJ)	7131 (2540)	6867 (2421)	7308 (2592)
Carbohydrate (g)	199 (78)	192 (77)	203 (77)
Fat (g)	69 (29)	65 (25)	72 (31)
Protein (g)	75 (28)	53 (11)	90 (26)
Protein intake adjusted for body mass (g/kg BM)	1⋅16 (0⋅46)	0⋅77 (0⋅15)	1⋅41 (0⋅41)

kJ, kilo joules (1 kcal = 4⋅186kJ); g, grams; g/kg BM, grams per kilogram of body mass.

### Performance of the protein screener

**Fig. 3.**
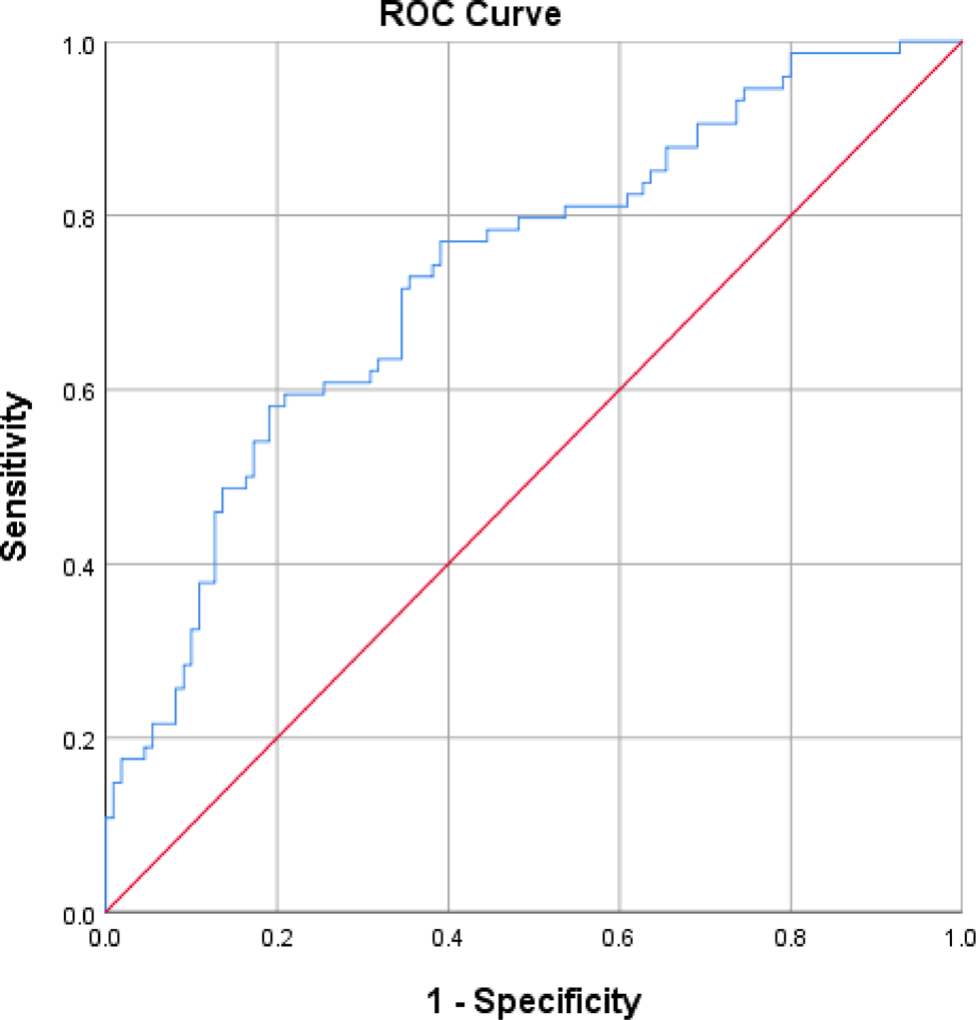
Receiver operation characteristic curve graph for the validation sample of all participants (*N* 184) to demonstrate the predictive value of the ProScreenerUK to discriminate between dichotomised protein intakes of ≤1⋅0 g/kg adjusted BM/d or >1⋅0 g/kg adjusted BM/d protein consumers based on the FFQ responses.

The number of responses to each question of the protein screener tool is reported in the Supplementary material. A ROC curve analysis was conducted to evaluate the predictive value of the ProScreenerUK to discriminate between dichotomised protein intakes of ≤1⋅0 g/kg adjusted BM/d or >1⋅0 g/kg adjusted BM/d protein consumers based on the FFQ responses. The AUC, which can be quantitatively used to define the accuracy of the test, was 0⋅731 (95 % CI 0⋅657, 0⋅805) with a standard error of 0⋅38, indicative of a ‘fair’ discriminative ability of the tool; 0⋅5 corresponds to no accuracy and 1⋅0 represents perfect accuracy^(29)^ (See Fig. 3). The number of cases in each cell, sensitivity and specificity, PPV and NPV is described for the probability cut-offs 0⋅1, 0⋅2 and 0⋅3 (Table 2). Based on the Youden index, the optimal cut-off was >0⋅1. Fifty-four out of 184 (30⋅4 %) participants were predicted by the protein screener tool to have protein intakes of ≤1 g/kg adjusted BM/d at the cut-off value 0⋅1, of these eighteen did not have protein intakes of ≤1 g/kg adjusted BM/d as derived from their FFQ responses. Seventy-four of the 184 (40⋅2 %) participants had a derived protein intake of ≤1 g/kg adjusted BM/d based on their FFQ responses. Thirty-eight participants were not captured by the protein screener as having protein intakes of ≤1 g/kg adjusted BM/d at a cut-off of ≤0⋅1, despite the FFQ data suggesting their dietary protein intakes were below this level. In total, thirty-six participants were identified as having protein intakes of ≤1 g/kg adjusted BM/d by both the protein screener and their FFQ responses.

**Table 2.** Sensitivity and specificity, PPV, NPV and Youden index for the protein screener against the FFQ outputs at the probability cut-offs 0⋅1, 0⋅2 and 0⋅3

(*N* 184)						
	Probability:					
	≤0⋅1	>0⋅1	≤0⋅2	>0⋅2	≤0⋅3	>0⋅3
Protein intake **>1⋅0** g/kg adjusted BM/d	*n* 92	*n* 18	*n* 104	*n* 6	*n* 108	*n* 2
Protein intake **≤1⋅0** g/kg adjusted BM/d	*n* 38	*n* 36	*n* 58	*n* 16	*n* 61	*n* 13
**Cut-off probability**	**>0⋅1**		**>0⋅2**		**>0⋅3**	
Sensitivity, % (95 %CI)	48⋅6 (48⋅3, 49⋅0)		21⋅6 (21⋅3, 21⋅9)		17⋅6 (17⋅3, 17⋅8)	
Specificity, % (95 % CI)	83⋅6 (83⋅4, 83⋅9)		95⋅5 (94⋅4, 94⋅7)		98⋅2 (98⋅1, 98⋅3)	
PPV, % (95 % CI)	66⋅7 (66⋅3, 67⋅1)		72⋅7 (72⋅1, 73⋅3)		86⋅7 (86⋅1, 87⋅2)	
NPV, % (95 % CI)	70⋅8 (70⋅5, 71⋅0)		64⋅2 (64⋅0, 64⋅4)		63⋅9 (63⋅7, 64⋅1)	
Youden index, % (95 %CI)	0⋅32 (0⋅25, 0⋅39)		0⋅17 (0⋅12, 0⋅22)		0⋅16 (0⋅11, 0⋅21)	

>, greater than; ≤, less than or equal to; g/kg adjusted BM/d, grams per kilogram adjusted body mass per day; PPV, Positive Predictive Value; NPV, Negative Predictive Value; CI, Confidence Interval, Youden Index; sensitivity + specificity −1.

## Discussion

The performance of the screener, based on an AUC of 0⋅731, suggests that it is applicable to identify if an individual is likely to consume protein intakes that are ≤1⋅0 g/kg BM/d; as such the tool has the potential capacity to screen for individuals who consume protein below the level of 1 g/kg (adjusted) BM/d.

This is the first rapid protein screening tool that has been validated in a UK population, across a range of ages. Previously developed short food questionnaires include screening for fat^(34,35)^ and cholesterol^(34)^ intake, which have been applied to identify individuals at risk of cardiovascular disease and metabolic disorders. Likewise, the Malnutrition Universal Screening Tool (MUST) is now widely used in NHS and care settings in the UK^(36)^. This is now a well-regarded tool to detect malnutrition and the early detection of individuals at risk of malnutrition allowing early intervention, demonstrating the value of screening tools.

ProScreenerUK has the advantageous features that it is convenient and inexpensive to implement and can be delivered remotely. This means that it may be used quickly and frequently in the population to screen for individuals who may warrant further investigation from healthcare professionals, such as dietitians. Low skeletal muscle mass has been linked to an increased risk of developing metabolic syndrome^(37)^ and cardiovascular disease (CVD)^(38)^ and low protein intakes may contribute to lower skeletal muscle mass. As such, monitoring protein intakes across the life course may limit metabolic syndrome risk^(39)^

In older adults, protein screening could prove to be beneficial to aid with understanding and addressing the levels of protein they should consume to prevent illnesses such as sarcopenia and osteoporosis^(2)^. Moreover, skeletal muscle reserves built during early adulthood can influence muscle loss rates experienced during later life^(40)^, demonstrating that adequate intakes of protein throughout the life course are evident. Further work will be needed to assess the tool's performance against the empirical benchmarks for a diagnostic instrument prior to adoption into care pathways. However, the tool may have secondary applications within the scientific community, particularly in the recruitment of individuals who may benefit the most from intervention studies based on their habitual protein intakes.

Analysis of the sensitivity-specificity (Table 2) demonstrates that this tool is most sensitive at a cut-off of 0⋅1, as supported by the Youden index score. Increasing the cut-off point leads (logically) to sensitivity being decreased, whilst specificity increases. A trade-off between these two is required and the decision about which cut-off to utilise should often be made in relation to the purpose on the screener^(29)^. Due to this tool being developed for screening purposes, it is most important to not miss a positive case (i.e. to not miss a case when an individual is likely to consume protein ≤1 g/kg BM/d); therefore, for this purpose, prioritising sensitivity over specificity is considered favourable. The optimum cut-off in this cohort differed from the one calculated in the Dutch sample of >0⋅3. This is likely due to the variation in validation methods and different approaches taken. The Dutch group utilised existing FFQ datasets against which the protein screener was validated, whereas our protocol required participants to complete both the protein screener and FFQ to allow a head to head comparison of protein intake using the two methods, thereby testing the real-world application of the tool. It is also possible that in the real-world individuals may overestimate their protein intake using the screener and this may provide another explanation for the differences observed in optimal cut-off points.

Due to national COVID-19 lockdown restrictions coinciding with this research, most of the data were collected by online survey responses. Although consideration was paid to gain responses from those who did not have access to the internet, by also collecting responses over the telephone, this still likely skewed our sample to a younger age group (Fig. 2). In total, forty-five individuals were over the age of 65 years of age. These numbers are too low to carry out ROC calculations using this subset of participants alone, so to further validate this screener specifically in an older adult population, an increase in the number of responses from this age category would be required. Seeking ways to engage these individuals in research of this kind is also warranted.

A key limitation of the validation was cross-referencing against an FFQ rather than the gold standard protocol of urinary excretion of nitrogen or a more accurate quantitative assessment instrument such as a weighed food diary or repeated 24-h recalls. In a validation of dietary assessment methods study, using 24-h urine nitrogen and potassium biological markers as the control comparison index, Bingham *et al.*^(41)^ demonstrated that the EPIC FFQ tool and 24-h recalls correlated the lowest with 24-h urine excretion and dietary N (0⋅10–0⋅27) compared to 7-d estimated food diaries (0⋅60–0⋅70) and 16-d of weighed records (0⋅78–0⋅87.) Future studies will address this by re-validating the tool using a more robust dietary intake collection methodology. However, as the tool is for screening rather than diagnosis, we argue that it is suitable for that specific application. Additional refinement and validation of this tool to maximise its ability to accurately identify individuals with low protein intakes would also benefit from validation with a greater number of individuals, including analyses by age ranges.

## Conclusion

A rapid (ten question) protein screener tool has been adapted and validated for use in an adult UK population to identify subjects who likely consume protein intakes of ≤1 g/kg BM.

## Data Availability

Data tables are presented in the published articles and supplementary material. Original data are available on request.

## Supplementary material

For supplementary material accompanying this paper visit https://doi.org/10.1017/jns.2022.96. click here to view supplementary material
