# Supplementary material for: Adaptation and validation of a protein intake screening tool for a UK adult population
Source: J Nutr Sci. 2022 Nov 10;11:e100. doi: 10.1017/jns.2022.96 (PMC9672832; doi:10.1017/jns.2022.96)
Supplement: Supplementary file 1 [file S2048679022000969sup001.docx]

Adaptation and validation of a protein screener in a UK adult population.

Esme R. Tuttiett^1,5,^, Elysa Ioannou^2^, Hanneke A.H. Wijnhoven^3^, Bernard M. Corfe^1,4^, Elizabeth A. Williams^1^

1. Department of Oncology and Metabolism, The Medical School, The University of Sheffield, Sheffield S10 2RX, UK
2. Sport and Physical Activity Research Centre, Sheffield Hallam University, Sheffield, S10 2BP, UK
3. Department of Health Sciences, Faculty of Science, and Amsterdam Public Health Research Institute, Vrije Universiteit Amsterdam, 1081 HV Amsterdam, The Netherlands.
4. Human Nutrition Research Centre, Faculty of Medical Sciences, Population Health Sciences Institute, Newcastle University, Newcastle NE2 4HH, UK

5. Author to whom correspondence should be addressed: Esme Tuttiett, University of Sheffield Medical School, Beech Hill Road, S10 2RX, email: [ertuttiett1@sheffield.ac.uk](mailto:ertuttiett1@sheffield.ac.uk), ORCID ID: https://orcid.org/0000-0002-7591-4099, phone: 0114 222 5522.

Responses to the Protein Screener questions (%) for participants of the UK validation study.

This table documents the responses provided to the protein screener questionnaire, expressed as percentage (%) of answers given for all participant (n = 184)

| **Question** | **Number of responses (%)** |
| --- | --- |
| Bread | |
| < 3 slices | 148 (80.4) |
| 3 slices | 11 (6.0) |
| 4 slices | 14 (7.6) |
| ≥ 5 slices | 10 (5.4) |
| Milk | |
| < 1 glass | 49 (26.6) |
| 1 glass | 81 (44.0) |
| ≥ 2 glasses | 54 (29.3) |
| Meat or meat substitute | |
| Small portion | 55 (29.9) |
| Medium portion | 128 (69.6) |
| Big portion | 1 (0.5) |
| Dairy Products | |
| <1 day/week | 58 (31.5) |
| 1 day/week | 13 (7.1) |
| 2 days/week | 22 (12.0) |
| 3 days/week | 16 (8.7) |
| 4 days/week | 18 (9.8) |
| 5 days/week | 11 (6.0) |
| 6 days/week | 13 (7.1) |
| 7 days/week | 33 (17.9) |
| Eggs |  |
| <1 day/week | 58 (31.5) |
| 1 day/week | 30 (16.3) |
| 2 days/week | 40 (21.7) |
| ≥3 days/week | 56 (30.4) |
| Pasta | |
| ≤1 day/4 weeks | 42 (22.8) |
| 2-3 days/4 weeks | 50 (27.2) |
| 1 day/week | 45 (24.5) |
| ≥1 day/week | 47 (25.5) |
| Fish | |
| ≤1 day/4 weeks | 31 (16.8) |
| 2-3 days/4 weeks | 18 (9.8) |
| 1 day/week | 49 (26.6) |
| ≥2 days/week | 86 (46.7) |
| Nuts | |
| <1 day/4 weeks | 26 (14.1) |
| 1-3 days/4 weeks | 45 (24.5) |
| ≥1 day/week | 113 (61.4) |
| Cheese | |
| <1 day/week | 35 (19.0) |
| 1 day/week | 21 (11.4) |
| 2 days/week | 33 (17.9) |
| 3 days/week | 32 (17.4) |
| 4 days/week | 20 (10.9) |
| 5 days/week | 16 (8.7) |
| 6 days/week | 10 (5.4) |
| 7 days/week | 17 (9.2) |
| Legumes (instead of slices of bread with cheese) | |
| <1 day/week | 46 (25.0) |
| 1 day/week | 26 (14.1) |
| 2 days/week | 39 (21.2) |
| 3 days/week | 35 (19.0) |
| 4 days/week | 13 (7.1) |
| 5 days/week | 18 (9.8) |
| 6 days/week | - |
| 7 days/week | 7 (3.8) |

**Syntax for calculating protein screener protein probability prediction scores.**

This demonstrates the coding inputted into SPSS to obtain predicted propertily scores for indivdual’s protein intakes based on the responses provided tot he protein screener.

** Encoding: UTF-8.*

**Syntax adaptation protein screener to UK questionnaire for XXX - XXX - August 2020.*

** get data set.*

** select analytical sample.*

*** Protein intake expressed in grams per kg bodyweight per day.*

** New variable: Adjusted body weight (based on paper of Berner et al. 2013).*

**calculate (adjusted) body weight.*

*compute BMI = weight/ (height_m * height_m).*

*execute.*

*IF (BMI < 18.5 & mfage < 71) weight_adj=(height_m) * (height_m) * 18.5.*

*EXECUTE.*

*IF (BMI > 25.0 & mfage < 71) weight_adj=(height_m) * (height_m) * 25.*

*EXECUTE.*

*IF (BMI >= 18.5 & BMI <= 25 & mfage < 71) weight_adj=weight.*

*EXECUTE.*

*IF (BMI < 22.0 & mfage >= 71) weight_adj=(height_m) * (height_m) * 22.*

*EXECUTE.*

*IF (BMI > 27.0 & mfage >= 71) weight_adj=(height_m) * (height_m) * 27.*

*EXECUTE.*

*IF (BMI >= 22.0 & BMI <= 27.0 & mfage >= 71) weight_adj=weight.*

*EXECUTE.*

** syntax for recoding original questions.*

**Variable names are based on the LASA dataset.*

** recode slices of bread (N05b_0).*

*RECODE N05b_0 (1 thru 3 = 1) (4=2) (5=1) (6 thru highest=1) (MISSING=SYSMIS) INTO*

*amount_slice_breadd1.*

*EXECUTE.*

*RECODE N05b_0 (1 thru 3 = 1) (4=1) (5=2) (6 thru highest=1) (MISSING=SYSMIS) INTO*

*amount_slice_breadd2.*

*EXECUTE.*

*RECODE N05b_0 (1 thru 3 = 1) (4=1) (5=1) (6 thru highest=2) (MISSING=SYSMIS) INTO*

*amount_slice_breadd3.*

*EXECUTE.*

** recode glasses of milk (N15b_0).*

*RECODE N15b_0 (1=1) (2=2) (3 thru highest=1) (ELSE=SYSMIS) INTO*

*amount_milkd1.*

*EXECUTE.*

*RECODE N15b_0 (1=1) (2=1) (3 thru highest=2) (ELSE=SYSMIS) INTO*

*amount_milkd2.*

*EXECUTE.*

**recode amount meat warm meal (N34b).*

** nb. option 0 = no meat.*

*RECODE N34b (0=1) (1=1) (2=2) (3=1) (4=1) (5=1) (ELSE=SYSMIS) INTO*

*amount_meatd1.*

*EXECUTE.*

*RECODE N34b (0=1) (1=1) (2=1) (3=2) (4=2) (5=2) (ELSE=SYSMIS) INTO*

*amount_meatd2.*

*EXECUTE.*

**recode frequency egg intake (N14a_0).*

*RECODE N14a_0 (1=1) (2=1) (3=1) (4=2) (5=1) (6 thru highest=1) (MISSING=SYSMIS) INTO*

*freq_eggd1.*

*EXECUTE.*

*RECODE N14a_0 (1=1) (2=1) (3=1) (4=1) (5=2) (6 thru highest=1) (MISSING=SYSMIS) INTO*

*freq_eggd2.*

*EXECUTE.*

*RECODE N14a_0 (1=1) (2=1) (3=1) (4=1) (5=1) (6 thru highest=2) (MISSING=SYSMIS) INTO*

*freq_eggd3.*

*EXECUTE.*

** recode frequency dairy dessert (N18a_0).*

*RECODE N18a_0 (1=1) (2=1) (3=1) (4=2) (5=3) (6=4) (7=5) (8=6) (9=7) (10=8) (MISSING=SYSMIS) INTO*

*freq_dairy_dessert.*

**recode frequency pasta (N24a_0).*

*RECODE N24a_0 (1=1) (2=1) (3=2) (4=1) (5 thru highest=1) (MISSING=SYSMIS) INTO*

*freq_pastad1.*

*EXECUTE.*

*RECODE N24a_0 (1=1) (2=1) (3=1) (4=2) (5 thru highest=1) (MISSING=SYSMIS) INTO*

*freq_pastad2.*

*EXECUTE.*

*RECODE N24a_0 (1=1) (2=1) (3=1) (4=1) (5 thru highest=2) (MISSING=SYSMIS) INTO*

*freq_pastad3.*

*EXECUTE.*

**recode frequency fish (N33a_0).*

*RECODE N33a_0 (1=1) (2=1) (3=2) (4=1) (5 thru highest=1) (MISSING=SYSMIS) INTO*

*freq_fishd1.*

*EXECUTE.*

*RECODE N33a_0 (1=1) (2=1) (3=1) (4=2) (5 thru highest=1) (MISSING=SYSMIS) INTO*

*freq_fishd2.*

*EXECUTE.*

*RECODE N33a_0 (1=1) (2=1) (3=1) (4=1) (5 thru highest=2) (MISSING=SYSMIS) INTO*

*freq_fishd3.*

*EXECUTE.*

** recode frequency peanuts (N62a_0).*

*RECODE N62a_0 (1=1) (2=2) (3=2) (4 thru highest =1) (MISSING=SYSMIS) INTO*

*freq_peanutsd1.*

*RECODE N62a_0 (1=1) (2=1) (3=1) (4 thru highest =2) (MISSING=SYSMIS) INTO*

*freq_peanutsd2.*

**recode frequency cheese on bread (N08a_0).*

*RECODE N08a_0 (1=1) (2=1) (3=1) (4=2) (5=3) (6=4) (7=5) (8=6) (9=7) (10=8) (MISSING=SYSMIS) INTO*

*freq_cheese_on_bread.*

*EXECUTE.*

** question in original screener, now replaced by legumes.*

**recode amount bread with cheese (N08b_0).*

*RECODE N08b_0 (1=1) (2=1) (3=2) (4 thru highest = 3) (MISSING=SYSMIS) INTO*

*amount_bread_with_cheese.*

*EXECUTE.*

*RECODE N08b_0 (1=1) (2=1) (3=2) (4 thru highest = 1) (MISSING=SYSMIS) INTO*

*amount_bread_with_cheesed1.*

*EXECUTE.*

*RECODE N08b_0 (1=1) (2=1) (3=1) (4 thru highest = 2) (MISSING=SYSMIS) INTO*

*amount_bread_with_cheesed2.*

*EXECUTE.*

** recode legumes question (named N26a_0 in the LASA dataset.*

** first recoding step (to freq_legumes) could be deleted if you like, it is not used in the model.*

*RECODE N26a_0 (1=1) (2=2) (3=3) (4 thru highest=4) (MISSING=SYSMIS) INTO*

*freq_legumes.*

*EXECUTE.*

*RECODE N26a_0 (1=1) (2=2) (3=1) (4 thru highest=1) (MISSING=SYSMIS) INTO*

*freq_legumesd1.*

*EXECUTE.*

*RECODE N26a_0 (1=1) (2=1) (3=2) (4 thru highest=1) (MISSING=SYSMIS) INTO*

*freq_legumesd2.*

*EXECUTE.*

*RECODE N26a_0 (1=1) (2=1) (3=1) (4 thru highest=2) (MISSING=SYSMIS) INTO*

*freq_legumesd3.*

*EXECUTE.*

**final model ORIGINAL protein screener with adjustment for shrinkage factor 0.92.*

**please check if the syntax works properly in at least 10 test cases, and randomly select some answer options for the variable on amount bread with cheese.*

**compare the resulting predicted probability score with the score from the online tool. These numbers should be the same.*

*COMPUTE z = 0.92*19.361 +0.106*0.92*weight_adj -0.326*0.92*amount_slice_breadd1 -1.175*0.92*amount_slice_breadd2*

*-2.750*0.92*amount_slice_breadd3 -0.344*0.92*amount_milkd1 -1.681*0.92*amount_milkd2*

*-1.326*0.92*amount_meatd1 -3.074*0.92*amount_meatd2 -0.175*0.92*freq_dairy_dessert*

*-0.256*0.92*freq_eggd1 -0.636*0.92*freq_eggd2 -1.480*0.92*freq_eggd3 -0.432*0.92*freq_pastad1*

*-0.713*0.92*freq_pastad2 -1.409*0.92*freq_pastad3 -0.454*0.92*freq_fishd1 -0.757*0.92*freq_fishd2*

*-1.100*0.92*freq_fishd3 -0.393*0.92*freq_peanutsd1 -0.888*0.92*freq_peanutsd2*

*-0.177*0.92*freq_cheese_on_bread -0.654*0.92*amount_bread_with_cheesed1 -1.214*0.92*amount_bread_with_cheesed2.*

*execute.*

**THIS IS THE FINAL MODEL AFTER VALIDATION IN THE HELIUS SAMPLE.*

**final model with adjustment for shrinkage factor 0.87 (!).*

**replace cheese on bread question by legumes question in final model.*

*COMPUTE z2 = 0.87*18.779 +0.104*0.87*weight_adj -0.383*0.87*amount_slice_breadd1 -1.305*0.87*amount_slice_breadd2*

*-2.906*0.87*amount_slice_breadd3 -0.256*0.87*amount_milkd1 -1.651*0.87*amount_milkd2*

*-1.277*0.87*amount_meatd1 -3.083*0.87*amount_meatd2 -0.163*0.87*freq_dairy_dessert*

*-0.219*0.87*freq_eggd1 -0.541*0.87*freq_eggd2 -1.357*0.87*freq_eggd3 -0.326*0.87*freq_pastad1*

*-0.592*0.87*freq_pastad2 -1.233*0.87*freq_pastad3 -0.489*0.87*freq_fishd1 -0.732*0.87*freq_fishd2*

*-1.063*0.87*freq_fishd3 -0.368*0.87*freq_peanutsd1 -0.838*0.87*freq_peanutsd2*

*-0.224*0.87*freq_cheese_on_bread -0.380*0.87*freq_legumesd1 -0.547*0.87*freq_legumesd2 -0.822*0.87*freq_legumesd3.*

*execute.*

**predicted probability score original screener.*

*COMPUTE predprob = 1/(1 + EXP(-z)) .*

*execute.*

**predicted probability score adapted screener with legumes question.*

*COMPUTE predprob2 = 1/(1 + EXP(-z2)) .*

*execute.*

**Regression equation and coding to go alongside syntax documentation.**

The following documents how the answers provided to the protein screener questionnaire are translated into SPSS inputs in ordered to go through the syntax simulation.


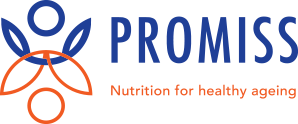


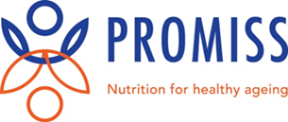


**Protein Screener older adults (Pro^55+^)**

**Author: XXX**

**10-10-2017 – syntax for XXX, SDU, Denmark**

**22-11-2019 – syntax for XXX, FAU Nürnberg, Germany**

**28-05-2020 - syntax for XXX, University of Sheffield, UK**

**Concept**


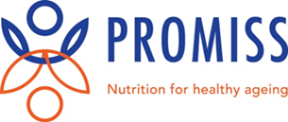
**Introduction text questionnaire**

The following questions are about your dietary habits. It is very important that you give an honest response. We would like to know what you ate or drank in the last 4 weeks (irrespective of week days, weekend days, at home or someplace else). If the last 4 weeks were very special (for example you were sick or you went on a vacation and this had a major influence on your usual diet), please recall the 4 weeks before this period.

These questions are about what you eat, not what another person in the household eats.

*(NB: the lay out of the questionnaire should be adapted for use in practice, the questionnaire below also includes information for research. Perhaps the answer categories should be in a horizontal line and not vertical and SPSS coding should be deleted)*

| *In the following questions, we ask how much of a food product you ate* |
| --- |

| SPSS variable  N05b_0 | 1. In the last 4 weeks, how many slices of bread did you eat on an average day? Mark one answer only. | | |
| --- | --- | --- | --- |
|  | SPSS coding  1  2  3  4  5  6  7  8  9  10  11  12  13  14 |  | Less than 1  1 slice  2 slices  3 slices  4 slices  5 slices  6 slices  7 slices  8 slices  9 slices  10 slices  11 slices  12 slices  > 12 slices |
| SPSS variable  N15b_0 | 2. In the last 4 weeks, how many glasses/cups of milk, buttermilk or soy milk did you drink on an average day? Mark one answer only. | | |
|  | SPSS coding  1  2  3  4  5  6  7  8  9  10  11  12  13  14 |  | Less than 1 glass  1 glass  2 glasses  3 glasses  4 glasses  5 glasses  6 glasses  7 glasses  8 glasses  9 glasses  10 glasses  11 glasses  12 glasses  > 12 glasses |

| *In the following questions, we will ask how often you ate a certain product* |
| --- |

| SPSS variable  N34b | 3. How much meat did you on average eat on 1 day that you ate meat with your warm meal in the last 4 weeks? Mark one answer only. **See picture** | | | | | | |
| --- | --- | --- | --- | --- | --- | --- | --- |
|  | SPSS coding  0  1  2  3  4  5 | | |  | Not applicable, does not eat meat  1/5 plate  1/4 plate  1/2 plate  2/3 plate  3/4 plate | | |
| SPSS variable  N18a_0 | | | 4. In the last 4 weeks how often did you yoghurt, quark, milk-based pudding, or soy dessert)? Mark one answer only. | | | | |
|  |  |  | SPSS coding  1  2  3  4  5  6  7  8  9  10 |  | | Not in these 4 weeks  1 day in 4 weeks  2 -3 days in 4 weeks  1 day/week  2 days/week  3 days/week  4 days/week  5 days/week  6 days/week  7 days/week | |
| SPSS variable  N14a_0 | | 5. In the last 4 weeks how often did you eat eggs with either your breakfast, lunch, evening meal, as a snack, or in a meal? Mark one answer only. | | | | | |
|  |  | SPSS coding  1  2  3  4  5  6  7  8  9  10 | |  | | | Not in these 4 weeks  1 day in 4 weeks  2 -3 days in 4 weeks  1 day/week  2 days/week  3 days/week  4 days/week  5 days/week  6 days/week  7 days/week |
| SPSS variable  N24a_0 | | 6. In the last 4 weeks how often did you eat pasta or noodles (like spaghetti, macaroni, lasagna, chow mein, rice-based or wheat-based noodles)? Mark one answer only. | | | | | |
|  |  | SPSS coding  1  2  3  4  5  6  7  8  9  10 | |  | | | Not in these 4 weeks  1 day in 4 weeks  2 -3 days in 4 weeks  1 day/week  2 days/week  3 days/week  4 days/week  5 days/week  6 days/week  7 days/week |

| SPSS variable  N33a_0 | 7. In the last 4 weeks how often did you eat fish with your bread meal, warm meal, or as a snack? (Do NOT include shellfish). Mark one answer only. | | |
| --- | --- | --- | --- |
|  | SPSS coding  1  2  3  4  5  6  7  8  9  10 |  | Not in these 4 weeks  1 day in 4 weeks  2 -3 days in 4 weeks  1 day/week  2 days/week  3 days/week  4 days/week  5 days/week  6 days/week  7 days/week |
| SPSS variable  N62a_0 | 8. In the last 4 weeks, how often did you eat nuts or peanuts as a snack? Mark one answer only. | | |
|  | SPSS coding  1  2  3  4  5  6  7  8  9  10 |  | Not in these 4 weeks  1 day in 4 weeks  2 -3 days in 4 weeks  1 day/week  2 days/week  3 days/week  4 days/week  5 days/week  6 days/week  7 days/week |

| SPSS variable  N08a_0 | 9. In the last 4 weeks how often did you eat cheese or cheese spread on your bread, bun, rusk, cracker, etc.? Mark one answer only. | | |
| --- | --- | --- | --- |
|  | SPSS coding  1  2  3  4  5  6  7  8  9  10 |  | Not in these 4 weeks  1 day in 4 weeks  2 -3 days in 4 weeks  1 day/week  2 days/week  3 days/week  4 days/week  5 days/week  6 days/week  7 days/week |
| SPSS variable  N08b_0 | 10. How many slices of bread, bun, rusk, cracker, etc. with cheese or cheese spread did you on average eat on a day that you ate cheese or cheese spread? Mark one answer only. | | |
|  | SPSS coding  1  2  3  4  5  6  7  8  9  10  11  12  13  14 |  | Less than 1  1 slice  2 slices  3 slices  4 slices  5 slices  6 slices  7 slices  8 slices  9 slices  10 slices  11 slices  12 slices  > 12 slices |

26a. Hoe vaak heeft u de laatste 4 weken **peulvruchten**, zoals bruine bonen, witte bonen, kidneybonen, kapucijners, linzen, kikkererwten etc. gegeten?

**Let op:** doperwten, tuinbonen, sperziebonen etc. en peulvruchten verwerkt in soep

niet meetellen.

| deze 4 weken niet | 1 dag  per 4 weken | | 2-3 dgn  per 4  weken | | 1 dag  per  week | | 2 dgn  per  week | | 3 dgn  per  week | | 4 dgn  per  week | | 5 dgn  per  week | | 6 dgn  per  week | 7 dgn  per  week | | |  |
| --- | --- | --- | --- | --- | --- | --- | --- | --- | --- | --- | --- | --- | --- | --- | --- | --- | --- | --- | --- |
| □ | □ | | □ | | □ | | □ | | □ | | □ | | □ | | □ | □ | |  |  |
| **↓**  **ga naar**  **vraag 27** |  |  | |  | |  | |  | |  | |  | |  | | |  | | |

26b. **Hoeveel** peulvruchten heeft u meestal op zo’n dag gegeten?

Het bord dat op de foto staat afgebeeld is een normaal dinerbord.


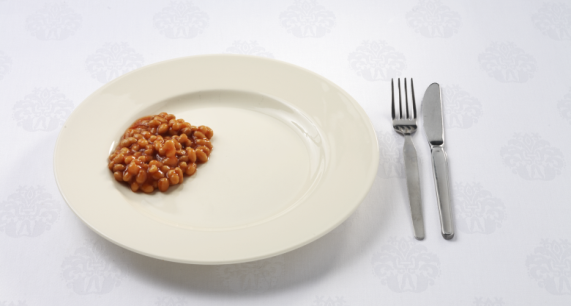
 □ 26-A
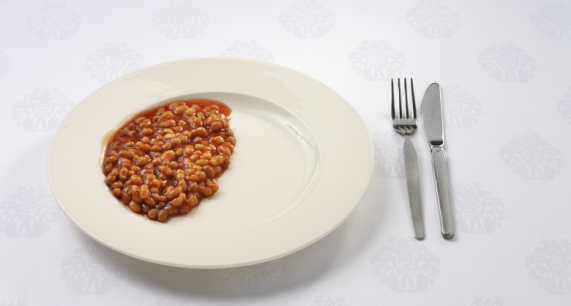
 □ 26-B


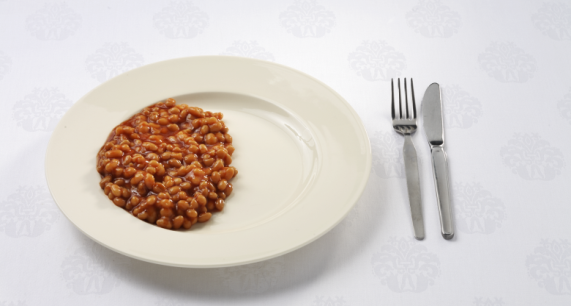
 □ 26-C
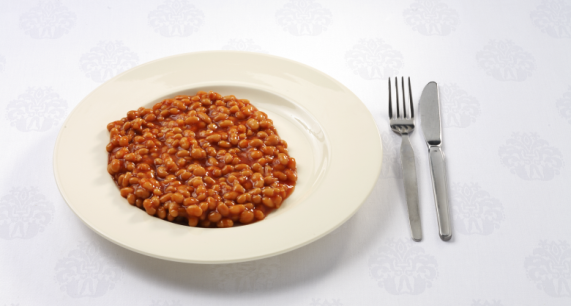
 □ 26-D


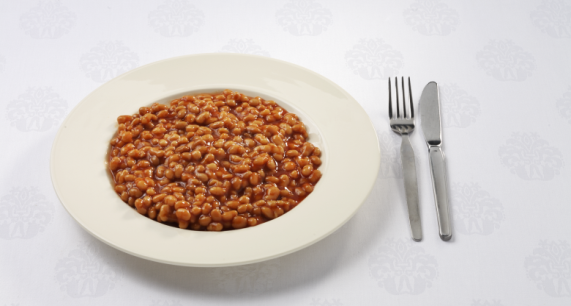
 □ 26-E

**Translation:**

In the last 4 weeks how often did you eat legumes, such as brown or white beans, kidney beans, capuchiners, lentils, ckickpeas? Please do not include peas, (green) beans, and legumes in soups. Mark one answer only.

How much legumes did you on average eat on a day that you ate legumes? (see pictures with a regular size dinner plate)
